# Effects of Physical Exercise on Telomere Length in Healthy Adults: Systematic Review, Meta-Analysis, and Meta-Regression

**DOI:** 10.2196/46019

**Published:** 2024-01-09

**Authors:** Juan Luis Sánchez-González, Juan Luis Sánchez-Rodríguez, Sergio Varela-Rodríguez, Rogelio González-Sarmiento, Cristina Rivera-Picón, Raúl Juárez-Vela, Clara Isabel Tejada-Garrido, Javier Martín-Vallejo, Víctor Navarro-López

**Affiliations:** 1 Faculty of Nursing and Physiotherapy University of Salamanca Salamanca Spain; 2 Department of Basic Psychology, Psychobiology and Methodology Faculty of Psychology University of Salamanca Salamanca Spain; 3 Molecular Medicine Unit, Instituto de Investigación Biomédica de Salamanca University of Salamanca-Sanidad de Castilla y León - Consejo Superior de Investigaciones Científicas Salamanca Spain; 4 Faculty of Health Sciences, Nursing Pontifical University of Salamanca Salamanca Spain; 5 Nursing Department Faculty of Heatlh Sciences University of La Rioja Logroño Spain; 6 Departament of Stadístics Faculty of Medicine University of Salamanca Salamanca Spain; 7 Department of Physical Therapy, Occupational Therapy, Rehabilitation and Physical Medicine Faculty of Health Sciences Universidad Rey Juan Carlos Madrid Spain

**Keywords:** meta-analysis, aging, exercise, older, telomere length

## Abstract

**Background:**

Physical exercise is one of the main nonpharmacological treatments for most pathologies. In addition, physical exercise is beneficial in the prevention of various diseases. The impact of physical exercise has been widely studied; however, existing meta-analyses have included diverse and heterogeneous samples. Therefore, to our knowledge, this is the first meta-analysis to evaluate the impact of different physical exercise modalities on telomere length in healthy populations.

**Objective:**

In this review, we aimed to determine the effect of physical exercise on telomere length in a healthy population through a meta-analysis of randomized controlled trials.

**Methods:**

A systematic review with meta-analysis and meta-regression of the published literature on the impact of physical exercise on telomere length in a healthy population was performed. PubMed, Cochrane Library, SCOPUS, Web of Science, and Embase databases were searched for eligible studies. Methodological quality was evaluated using the Risk Of Bias In Nonrandomized Studies of Interventions and the risk-of-bias tool for randomized trials. Finally, the certainty of our findings (closeness of the estimated effect to the true effect) was evaluated using Grading of Recommendations, Assessment, Development, and Evaluations (GRADE).

**Results:**

We included 9 trials that met the inclusion criteria with fair methodological quality. Random-effects model analysis was used to quantify the difference in telomere length between the exercise and sham groups. Meta-analysis showed that exercise did not significantly increase telomere length compared with the control intervention (mean difference=0.0058, 95% CI −0.05 to 0.06; *P*=.83). Subgroup analysis suggested that high-intensity interventional exercise significantly increased telomere length compared with the control intervention in healthy individuals (mean difference=0.15, 95% CI 0.03-0.26; *P*=.01). Furthermore, 56% of the studies had a high risk of bias. Certainty was graded from low to very low for most of the outcomes.

**Conclusions:**

The findings of this systematic review and meta-analysis suggest that high-intensity interval training seems to have a positive effect on telomere length compared with other types of exercise such as resistance training or aerobic exercise in a healthy population.

**Trial Registration:**

PROSPERO CRD42022364518; http://tinyurl.com/4fwb85ff

## Introduction

### Background

Telomeres are nucleoprotein structures located at the ends of eukaryotic chromosomes and are of critical importance both in the maintenance of genomic stability and in the processes of tumor suppression and aging [1]. In most eukaryotic cells, telomeres consist of tandem repeats of a guanine-rich sequence (TTAAGGG) that develop at the end of chromosomes in the 5' to 3' direction, with a complementary cytidine-rich chain [2]. Telomeric sequences may vary between species; however, every organism possesses the same repetitive sequence for all telomeres. At birth, the telomeres of human somatic cells contain approximately 15 kilobases. In the absence of telomerase, an average of 25 to 200 bases are lost from the telomeric ends at each cell division [3]; when the length of the telomere reaches below a critical limit, cell division ceases, and the cell ages and dies [4]. The main function of telomeres is to protect the ends of chromosomes and prevent their degradation and fusion while maintaining genomic stability [5,6].

Several studies have suggested that short telomere length is associated with progressive acceleration of aging, including an increase in age-related diseases such as osteoporosis, cancer, and dementia [7-9]. Therefore, it seems evident that controlling telomere length could be a key factor in the aging process and health care.

Regular physical exercise is one of the main nonpharmacological strategies used to prevent the onset of age-related diseases. Physical exercise is defined as planned, structured, repetitive, and purposeful physical activity, that is, for the improvement or maintenance of one or more components of physical fitness [10]. Werner et al [11] observed that endurance athletes have a larger telomere size than inactive controls. Moreover, physically active middle-aged twins have longer telomeres than inactive siblings [12]. Therefore, it seems clear that regular physical exercise is essential for healthy aging and supporting positive mental health. It can help delay, prevent, or manage many costly and difficult chronic diseases faced by older adults [13]. It can also reduce the risk of premature death and moderate or severe functional limitations in older adults [14].

However, few prospective studies have evaluated the effect of physical exercise on telomere length. In addition, these studies had large methodological differences, such as heterogeneous samples, different physical exercise modalities, and varied time and duration of the interventions. To date, only one meta-analysis [15] has studied the relationship between different physical exercise modalities and telomere length; however, the populations analyzed in the qualitative and quantitative analyses were heterogeneous. Therefore, the results should be cautiously interpreted.

### Objective

This systematic review and meta-analysis aimed to study the impact of different physical exercise modalities on telomere length in prospective studies (clinical trials and randomized controlled trials [RCTs]) in which the study sample comprised a healthy population without any type of pathology.

## Methods

This systematic review and meta-analysis was conducted in accordance with the guidelines of the PRISMA (Preferred Reporting Items for Systematic Reviews and Meta-Analyses; Multimedia Appendix 1) [16].

### Literature Search

To identify relevant studies on the impact of physical exercise on telomere length, we conducted a systematic literature search using the following English-language databases (until September 2022): PubMed, Web of Science, SCOPUS, Embase, and Cochrane Library. The search was performed independently by 2 researchers (JLSG and VNL). The search strategy used to identify all potential studies using the following terms is detailed in Multimedia Appendix 2 [17-25].

We also manually searched the references cited in selected articles or reviews to identify relevant studies.

### Study Selection

We used the Population, Intervention, Comparison, Outcomes, Time, and Study design as a framework to formulate eligibility criteria (Textboxes 1 and 2) [26].

**Population, Intervention, Comparison, Outcomes, Time, and Study design framework.**

- Population: healthy adults with no neurological disease
- Intervention: interventions with exercise as the main focus were selected
- Compare: control group not performing physical exercise
- Outcomes: telomere length was assessed using both peripheral blood and saliva samples
- Time: no temporal restrictions were applied to the duration of the intervention or outcome measures
- Studies: only randomized controlled trials (RCTs) and controlled trials were included

**Inclusion and exclusion criteria.**

**Inclusion criteria**

- Article type
- Randomized controlled trials or controlled trials
- Language
- English
- Population
- Healthy population
- Type of intervention
- Aerobic exercise, resistance training, or high-intensity interval training
- Outcome
- Measurement of telomere size by peripheral blood or saliva collection

**Exclusion criteria**

- Article type
- Case studies, systematic reviews, and meta-analyses
- Language
- Spanish, Chinese, French
- Population
- Population with neoplastic processes, neurodegenerative diseases, and cognitive alterations
- Type of intervention
- Any other type of nonexercise intervention
- Outcome
- Any other type of measure that purports to measure aging but is not telomeric length

### Data Extraction

Two investigators (JLSG and VNL) independently extracted data. A standardized methodology was used to obtain data from studies that met the inclusion criteria. Data were obtained for the first author, year of publication, design, patient characteristics, intervention protocol and timing, study outcomes (telomere size before and after intervention), and the telomere size calculation technique. In addition, the means and SDs of the study results were obtained. The authors of the included studies were contacted via email to access potentially unclear data. If no responses were received, the data were excluded from the analysis.

### Interrater Reliability

Interrater reliability for screening, risk of bias assessment, and quality of the evidence rating were assessed using percentage agreement and Cohen κ coefficient [27,28]. There was strong agreement between the reviewers for the screening records and full texts (98.51% agreement rate and κ=0.91) and the risk of bias assessment (92.86% agreement rate and κ=0.83).

### Risk of Bias and Assessment Methodological Quality of the Studies

Two reviewers independently assessed the risk of bias of the included studies (SVR and VNL).

The risk of bias in nonrandomized studies of interventions (NRSIs) was assessed using the Risk Of Bias In Nonrandomized Studies of Interventions (ROBINS-I) [29]. This tool assesses the risk of bias in NRSI results. The types of NRSIs that can be assessed with this tool are quantitative studies that estimate the efficacy (harm or benefit) of an intervention and did not use randomization to assign units (individuals or groups of individuals) to comparison groups. ROBINS-I considers 6 domains: randomization process (D1), bias arising from period and carryover effects (DS), deviations from the intended interventions (D3), missing outcome data (D4), and selection of the reported result (D5).

In contrast, a revised tool was used to assess the risk of bias in randomized clinical trials (risk-of-bias tool for randomized trials; RoB2) [30]. The tool was structured into 5 domains through which bias could be introduced into the outcome. These were identified on the basis of empirical evidence and theoretical considerations. Because the domains cover all types of bias that may affect the results of randomized trials, each domain is mandatory; and no additional domains should be added. The 5 domains for individual randomized trials (including crossover trials) were bias arising from the randomization process (D1), bias due to deviations from intended interventions (D2), bias due to missing outcome data (D3), bias in measurement of the outcome (D4), and bias in selection of the reported result (D5) [31,32].

### Overall Quality of Evidence

The overall quality of evidence was based on classifying the results into levels of evidence according to the Grading of Recommendations Assessment, Development, and Evaluation (GRADE), which is based on five domains: (1) study design, (2) imprecision, (3) indirect, (4) inconsistency, and (5) publication bias.

Evidence was categorized into the following four levels accordingly: (1) high quality: further research is very unlikely to change our confidence in the estimate of effect, and all 5 domains are also met; (2) moderate quality: further research is likely to have an important impact on our confidence and might change the estimate of effect, and 1 of the 5 domains is not met; (3) low quality: further research is very likely to have an important impact on our confidence and is likely to change the estimate of effect, and 2 of the 5 domains are not met; and (4) very low quality: any estimate of effect is very uncertain, and 3 of the 5 domains are not met [31,32].

### Statistical Analysis

Mean differences (MDs) after the intervention were used to compare values between the exercise and control groups, with a 95% CI. To obtain the effect size, the MD between the groups was converted to the standardized MD with a 95% CI. Statistical significance was set at *P*<.05. The individual effect size of each study and calculation of the overall effect size are presented as forest plots.

The restricted maximum likelihood method estimated the variance of between-study heterogeneity; the presence of between-study heterogeneity was assessed with the Cochran Q statistic test (with *P*<.05 considered significant) and the degree of heterogeneity with the inconsistency index (*I*^2^) [33]. An *I*^2^ value between 0% and 25% was considered to represent small heterogeneity, between 25% and 75% medium heterogeneity, and >75% large heterogeneity [34]. *I*^2^ complements the *Q* test, although it has the same power problems when the number of studies is small [34]. When the *Q* test was significant (*P*<.10) and the *I*^2^ result was >25%, indicating heterogeneity between studies, the random-effects model was applied in the meta-analysis. When heterogeneity was >25% according to the *I*^2^ statistic, outliers (studies whose 95% CI cutoff was lower and greater than the pooled 95% CI upper and lower cutoff) and influential case analysis were performed using the analysis according to the graph of Baujat et al [35] (graph showing the contribution of each study to the overall heterogeneity compared with its contribution to the overall pooled result). The identified studies were flagged as outliers or influential cases and were removed. A subgroup analysis was performed according to the type of exercise used (resistance training, aerobic exercise, or high-intensity interval training [HIIT]). An a priori meta-regression analysis was performed on the variables of exercise intensity and duration, as well as the year of publication and methodological quality, to evaluate whether these variables influenced the overall effect size.

Skewness was assessed using a contour-enhanced funnel plot in analyses consisting of at least 5 studies, indicating the possible publication bias of small studies small studies with negative results. In the absence of publication bias, the plot resembled a symmetrical funnel-shape.

Studies were analyzed with R software (R Foundation for Statistical Computing) [36], using the Metafor package [37] as detailed by Harrer et al [38], and with the computer software Review Manager (version 4.1; The Cochrane Collaboration).

## Results

### Study Selection and Characteristics

**Figure 1:**
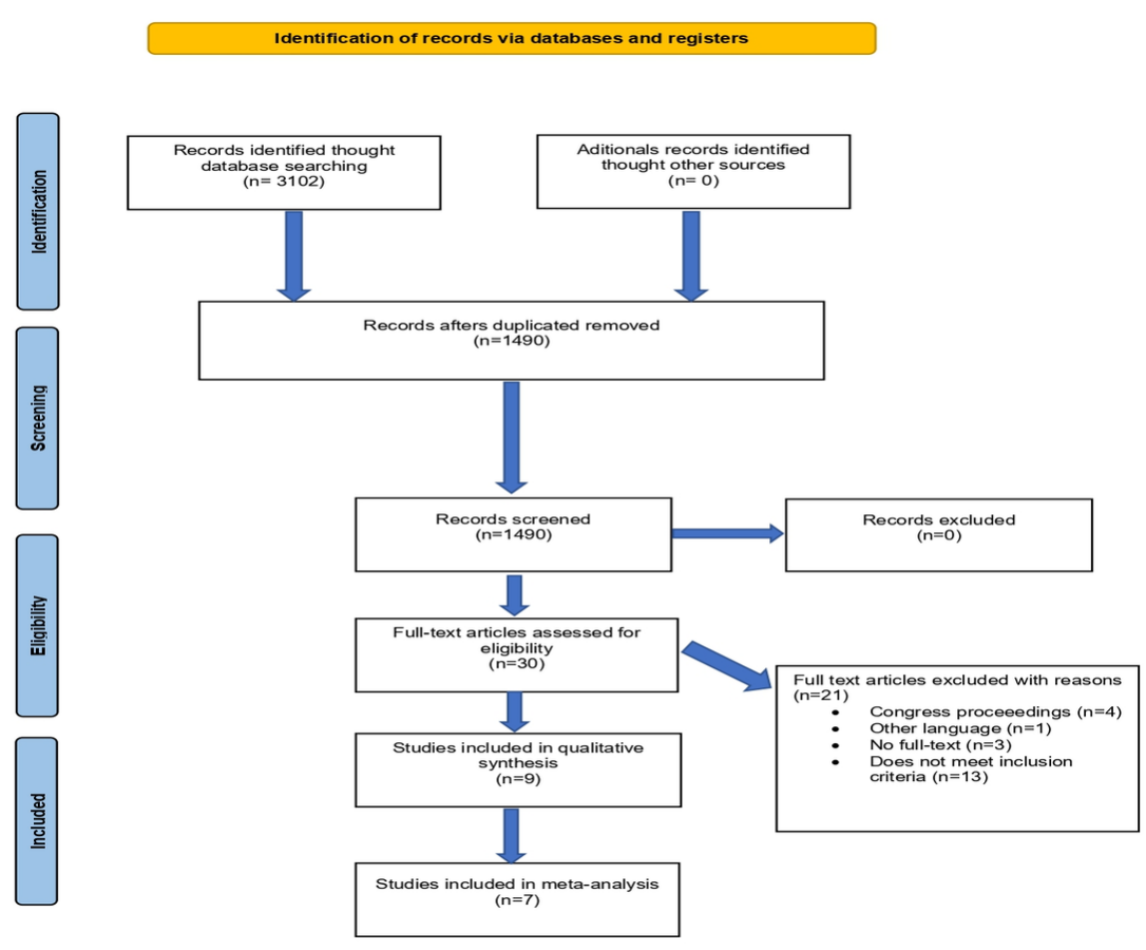
Flow diagram.

The search found 3102 records, of which 1612 were duplicates and 1490 were screened by title and abstract. We found 30 studies that were potentially relevant and excluded 21 studies after screening their full reports. Finally, 9 studies met the eligibility criteria and were included in the qualitative analysis, and 7 studies, which included 1320 participants, were included in the quantitative analysis. The entire screening process is shown in the PRISMA (Preferred Reporting Items for Systematic Reviews and Meta-Analyses) flow diagram in Figure 1.

### Qualitative Summary of the Included Studies

**Figure 2:**
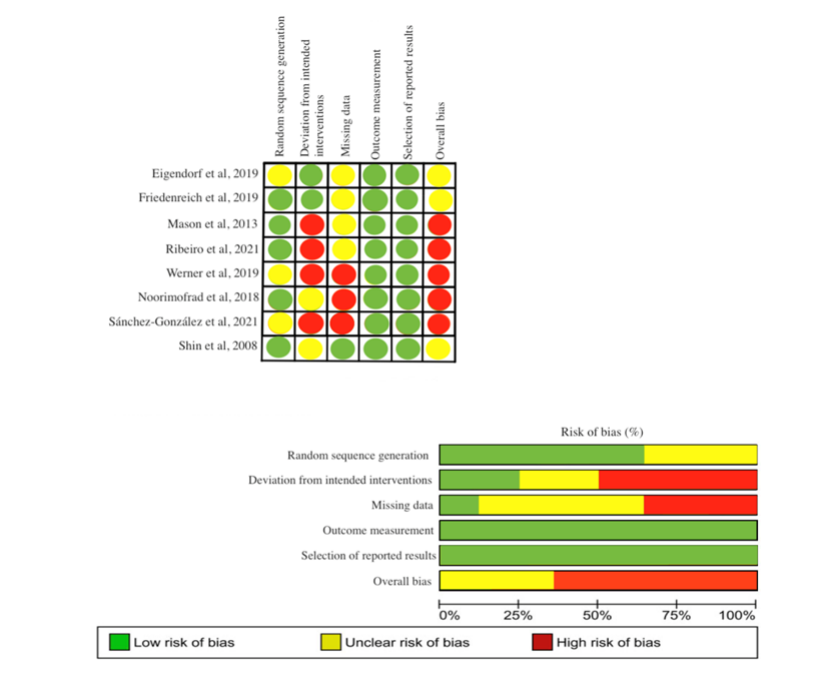
Risk of bias.

All 9 studies were intervention studies (RCTs or controlled trials) and were of fair to good methodological quality according to the ROBINS-I or RoB2 scale (Figure 2 [17-20,22-25]). These studies were conducted in Germany [17,18], Canada [19], the United States [20], the United Kingdom [21], Iran [22], Brazil [23], Spain [24] and South Korea [25]. A total of 1320 participants were included, including both men and women, with the latter being represented to a greater extent (98%). Regarding the type of exercise, 4 studies performed resistance training [17,18,21,25], 4 used aerobic exercises [17,19,20,23], 3 used HIIT [17,22,23], and 1 used combined training (aerobic plus resistance training) [24]. Regarding protocol duration, 1 study conducted a 2-week intervention [22], 1 study conducted a 4-week intervention [23], 4 studies conducted a 6-week intervention [17,18,24,25], and 3 studies conducted a 12-week intervention [19-21]. The intensity information for each protocol is presented in Table 1.

**Table 1:** Participant characteristics.

Study	Design	Group: sample size; age (y), mean (SD)			Sample size, n		Protocol intervention		Duration (wk)		Laboratory techniques and procedures		Quality score (PEDro^a^)
		Male	Female										
Noorimofrad and Ebrahim [22], 2018	RCT^b^	G1: 15; 19.7 (1.1) G2: 15; 20.13 (0.64)	G1: 0 G2: 0	30		G1: HIIT^c^. 6-10 laps of HIIT 30 s at an intensity of 150%-175% of Pmax. G2: control group		8		PCR^d^. The DNA from these cells was extracted using standard salting out- proteinase K method. The concentration and quality of the extracted DNA were examined using NanoDrop at wavelengths of 260 and 280 nm, and the ratio of the 2 wavelengths was used. Two PCR reactions were performed for each sample, the first reaction for telomeric DNA fragment and the second for its control gene, acid ribosomal phosphoprotein. DNA telomeric length was calculated based on the ratio of telomere to control gene.		4/11	
Ribeiro et al [23], 2021	RCT	G1: 0 G2: 0 G3: 0	G1: 30; 28.50 (5.76) G2: 28; 29.14 (5.26) G3: 29; 28.97 (4.32)	87		G1: control group G2: continuous aerobic physical training. Treadmill training, 3 times a week for 16 weeks, from 30 min in the first week to 50 min in the last week. G3: intermittent aerobic physical training. Treadmill training, 3 times a week for 16 weeks, from 30 min in the first week to 50 min in the last week		16		PCR. DNA integrity was accessed by agarose gel stained with GelRead, and the concentration was determined using the NanoDrop 2000c spectrophotometer. Telomere length was determined by calculating the telomere to single-copy gene ratio using DCt^e^(Ct[telomere]/Ct[single gene]). The telomere length was expressed as the relative T/S^f^ ratio, normalized to the mean of the T/S ratio of the reference sample		6/11	
Shin et al [25], 2008	RCT	G1: 8; (46.8 (6.4) G2: 8; 46.8 (6.4) G3: 0	G1: 0 G2: 0 G3: 15; 74.91 (5.45)	31		G1: aerobic exercise. 3 d/wk. 10 min warm-up, 45-min treadmill walk/run at 60% VO2max^g^, 5 min cool-down. G2: did not participate in any form of regular exercise. G3: control group		24		PCR. The final telomere oligo-primer concentration were tel1, 270 nM; tel 2, 900 nM. The final 36B4 (single copy gene oligo-primer concentrations were 36B4u, 300 nM; 36B4d, 500 nM. Relative T/S values were determined by sample T/S values compared with reference DNA T/S values		5/11	
Werner et al [17], 2019	RCT	G1: 12; 50.2 (7.4) G2: 9; 49.5 (7) G3: 10; 48.4 (6.5) G4: 14; 48.1 (7.5)	G1: 23; 50.2 (7.4) G2: 17; 49.5 (7) G3: 19; 48.4 (6.5) G4: 20; 48.1 (7.5)	114		G1: control group G2: aerobic endurance training. 3 d/wk, 45-min session G3: interval training. 3 d/wk, 45-min session G4: resistance training. 3 d/wk, 45-min session		24		PCR. DNA concentrations were quantified photometrically to ensure sufficient quantity and purity. PCR data were exported to Microsoft Excel, formatted, and analyzed with the comparative Ct method (2-ΔΔCt) to calculate T/S ratios and thereby relative differences in the amount of telomere repeat DNA between the individual pre- vs poststudy time points		5/11	
Sánchez-González et al [24], 2021	RCT	G1: 0 G2: 0 G3: 9; 49.5 (7) G4: 10; 48.4 (6.5) G5: 14; 48.1 (7.5)	G1: 41; 72.70 (4.13) G2: 33; 71.21 (4.32) G3: 17; 49.5 (7) G4: 19; 48.4 (6.5) G5: 20; 48.1 (7.5)	74		G1: aerobic+resistance training. 3 d/wk. G2: control group G3: aerobic endurance training. 3 d/wk, 45-min session G4: interval training. 3 d/wk, 45-min session G5: resistance training. 3 d/wk, 45-min session		24		PCR. DNA was determined by measuring the absorbance at 260 nm using a NanoDropTM 2000/2001 spectrophotometer. The purity of the DNA was analyzed based on the A260/280 absorbency ratio, where an optimal purity ratio ranged between 1.8 and 2.0. The Ct comparative method was used to calculate the relative expression levels of each amplicon		4/11	
Eigendorf et al [18], 2019	RCT	G1: 0 G2: 0	G1: 146; 53.0 (4.9) G2: 145; 52.8 (4.7)	291		G1: resistance training. 210 min of resistance training per week for 6 months G2: control group		24		PCR. For assessment of telomere length, genomic DNA was extracted from peripheral blood mononuclear cells using QIA amp DNA Mini kit (Qiagen, Hilden, Germany). Telomere length was calculated as abundance of telomeric template vs a single copy gene (36B4)		7/11	
Nickels et al [21], 2022	CT^h^	G1: 0 G2: 0	G1: 11; 50.8 (7.5) G2: 11; 49.3 (6.1)	22		G1: pilates training. Minimum of 2 sessions of 1 h for 12 months. G2: control group		52		PCR. Whole blood was utilized as the starting material for DNA extraction and the concentration and purity were evaluated by spectrophotometry. Intra-assay coefficient of variation for calculated T/S ratio was 4.6%. Interassay coefficient of variation for calculated T/S ratio was 2.8%		4/11	
Mason et al [20], 2013	RCT	G1: 0 G2: 0 G3: 0 G4: 0	G1: 87; 58 G2: 118; 58 G3: 117; 58 G4: 117; 58	439		G1: did not receive intervention G2: calorie-reduced diet G3: aerobic exercise. 45-min moderate to vigorous (≥4 METs^i^) exercise at heart rate of 70%-85%, 5 d/wk. G4: aerobic exercise (45-min moderate to vigorous [≥4 METs] exercise at heart rate of 70%-85%, 5 d/wk)+calorie-reduced diet		52		PCR. The DNA from these cells was extracted using standard salting out-proteinase K method. The concentration and quality of the extracted DNA were examined using Nano drop at wavelengths of 260 and 280 nm, and the ratio of the 2 wavelengths were used. Two PCR reactions were performed for each sample, the first reaction for telomeric DNA fragment and the second for its control gene, acid ribosomal phosphoprotein. DNA telomeric length was calculated based on the ratio of telomere to control gene		4/11	
Friedenreich et al [19], 2019	RCT	G1: 0 G2: 0	G1: 99; 60.4 G2: 113; 60	212		G1: aerobic exercise ≥45 min for 5 d/wk (supervised 3 d/wk by ALPHA^j^ Trial exercise trainers+unsupervised 2 d/wk) Sedentary individuals (exercise <90 min/wk or 90–120 min/wk maximum)		52		PCR Sample reactions were set up in triplicate using the EpMotion 5075 (Eppendorf, United States), containing 20 ng of template DNA, Power SYBR Green PCR		8/11	

^a^PEDro: Physiotherapy Evidence Database.

^b^RCT: randomized controlled trial.

^c^HIIT: high-intensity interval training.

^d^PCR: polymerase chain reaction.

^e^DCt: delta cycle threshold.

^f^T/S: telomere/single gene.

^g^VO2max: Volume of Oxygen Maximum.

^h^CT: controlled trial.

^i^MET: metabolic equivalents.

^j^ALPHA: Alberta Physical Activity and Breast Cancer Prevention.

### Risk of Bias

Owing to the design of the included studies, 8 were analyzed using the RoB2, and 1 study was analyzed using the ROBINS-I. As assessed by the RoB2 and ROBINS-I, 56% (5/8) of the studies showed a high risk of bias, 33% (2/8) showed some concerns, and 11% (1/8) showed a low risk of bias. The item with the highest risk of bias was “deviations from the intended interventions” in which 45% (3/8) of the studies showed a high risk of bias, and the item “missing data” had 33% (2/8) of the studies that showed a high risk of bias in therapist blinding (Figure 2).

### Effects of Exercise on Telomere Length

**Figure 3:**
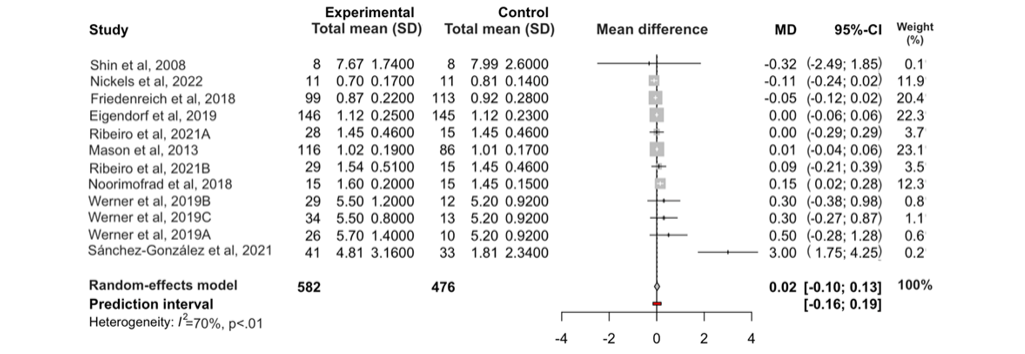
Forest plot of the effect of exercise on telomere length. Forest plot of the results of a random-effects meta-analysis is shown as mean differences with 95% CI for the comparison of mean telomere length in the exercise and control groups. The shaded square represents the point estimate for the individual study and the study weight in the high-intensity group. Diamond represents the overall mean difference between studies.

The meta-analysis showed that overall, exercise did not produce a significant increase in telomere length compared with that of the control groups, which did not exercise (MD 0.02, 95% CI −0.10 to 0.13; *P*=.77; N=1058; Figure 3 [17-25]). The restricted maximum likelihood method estimated a between-study heterogeneity variance of τ^2^=0.0034 and an *I*^2^ value of 70%, indicating significant heterogeneity among the studies included in the analysis (*P*<.01).

**Figure 4:**
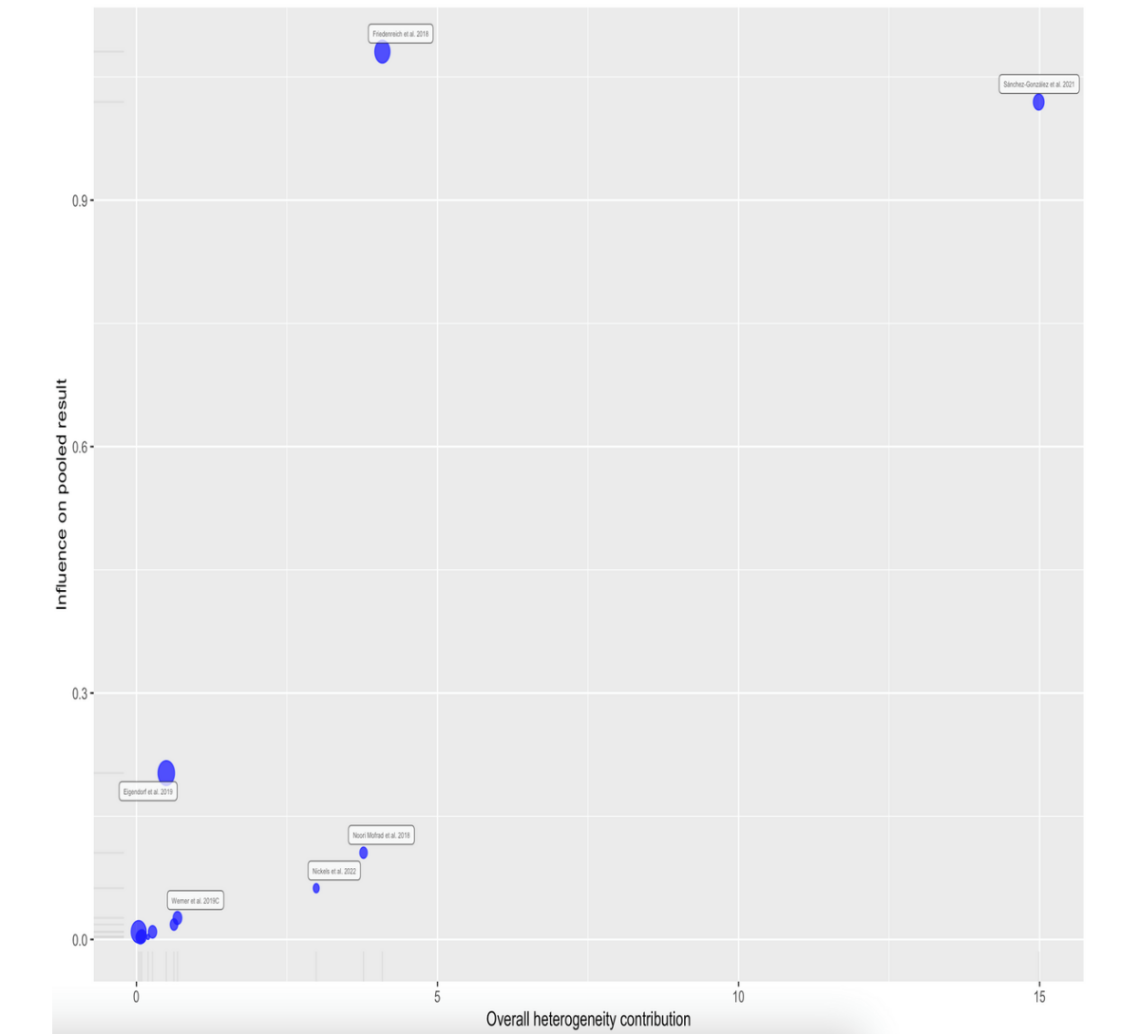
Funnel plot.

**Figure 5:**
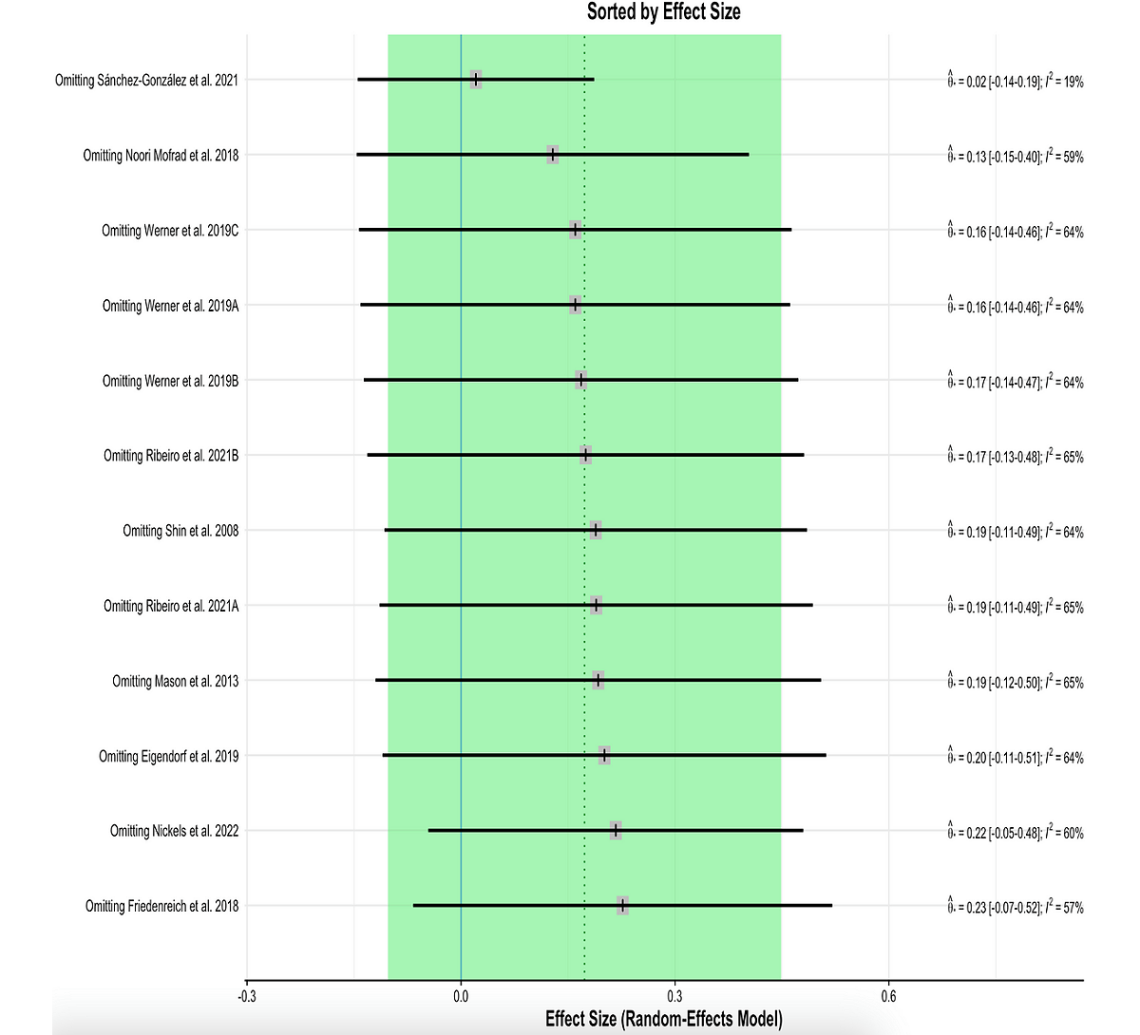
Influence of pooled result.

When performing an analysis of influential cases in the heterogeneity and outlier studies (random-effects model), we detected 2 influential cases (Figures 4 and 5) [17-25]: the study by Sánchez-González et al [24] (which was also considered an outlier) and the study by Friedenreich et al [19]. Excluding the influential cases from the meta-analysis resulted in reduced heterogeneity between studies (23%) and did not affect the results of the meta-analysis (Table 2).

**Figure 6:**
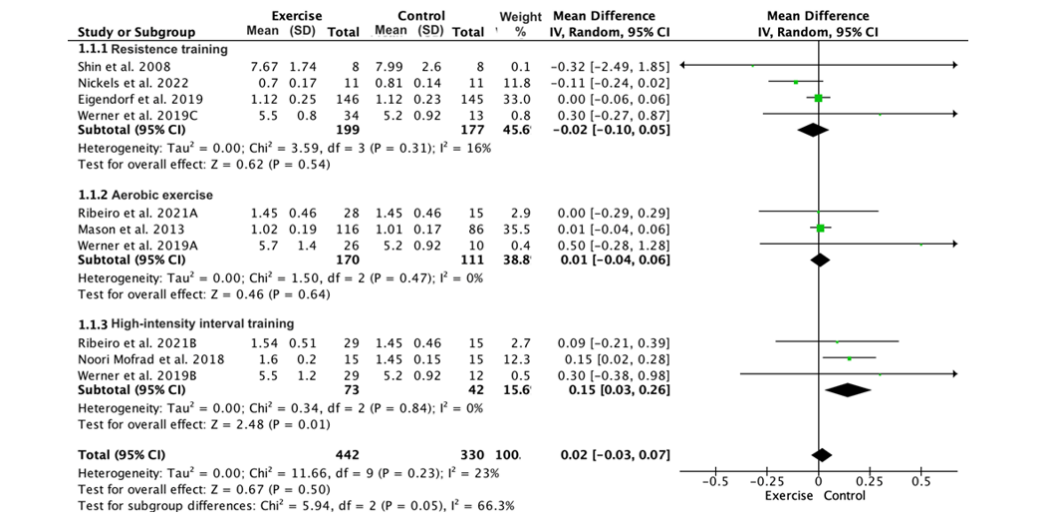
Subgroup analysis of the effect of exercise on telomere length. Forest plot of the results of a random-effects meta-analysis shown as mean differences with 95% CI for the comparison of mean telomere length in the exercise group and the control group, performing subgroup analyses for each type of exercise included (resistance training, aerobic exercise, and high-intensity interval training). The shaded square represents the point estimate for each study and the weight of the study in the meta-analysis. Diamond represents the overall mean difference between studies.

The subgroup analysis according to the type of exercise showed significant differences between the groups (*P*=.05). Resistance training (MD −0.02, 95% CI −0.01 to 0.05; *P*=.54; *I*^2^=16%) and aerobic exercise (MD −0.01, 95% CI −0.0 to 0.06; *P*=.64; *I*^2^=0%) groups showed no significant differences compared with the control group, but the HIIT group showed significant differences compared with the control group, with a greater telomere length observed in the HIIT group (MD 0.15, 95% CI 0.03-0.26; *P*=.01; *I*^2^=0%; Figure 6 [17,18,20-23,25]).

Meta-regression analysis showed no relationship between exercise intensity and duration, year of publication, and methodological quality of the included studies (*P*<.05).

**Table 2:** Meta-analysis without influential and outlier cases.

Analysis	MD^a^ (95% CI)	*P* value	Heterogeneity, *I*^2^ (%)
Main analysis	0.02 (−0.10 to 0.13)	.77	70
Outliers removed^b^	0.0058 (−0.05 to 0.06)	.83	30
Influential cases removed^c^	0.02 (−0.03 to 0.07)	.50	23

^a^MD: mean difference.

^b^Removed as outliers: Sánchez-González et al [24].

^c^Removed as influential studies: Sánchez-González et al [24] and Friedenreich et al [19].

### Analysis of Publication Bias

**Figure 7:**
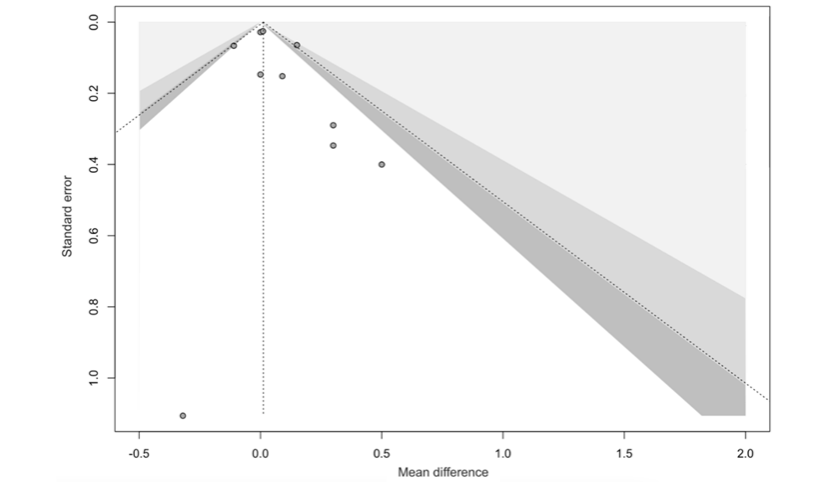
Contour-enhanced funnel plot of the included studies. Dispersion of effect size. x-axis: observed effect sizes. y-axis: inverse SE (higher values on the y-axis represent lower SEs). Slight asymmetry, indicating possible publication bias. Inside to outside (0-2). White region *P*>.05; dark gray region *P*<.10; intermediate gray region *P*≤.05; outer gray region *P*≤.001.

The contour-enhanced funnel plot showed asymmetry, which indicated heterogeneity among the included studies. Most of the studies included in this analysis were not significant; therefore, publication bias was ruled out (Figure 7).

### Quality of Evidence

Table 3 provides the details of the GRADE assessment. Three levels of evidence were downgraded due to the serious risk of bias and high heterogeneity (inconsistency) of the results, which suggests a very small level of evidence regarding the effects of overall physical exercise modalities on telomere length. In the subgroup analysis, inconsistency was rated as not serious for the 3 exercise modalities, and the level of evidence depended on the risk of bias, being moderate for resistance training and small for aerobic exercise and HIIT.

**Table 3:** Grading of Recommendations Assessment, Development, and Evaluation assessment.

Exercise modality, studies, and sample size			Risk of bias^a^		Inconsistency^b^		Indirectness^c^		Imprecision^d^		Publication bias^e^		MD^f^ (95% CI)		Quality of evidence
**Overall**															
	12 trials (n=1058)	Very serious (mainly by deviations from intended interventions and missing data)		Serious (*I*^2^=70%)		Not serious		Not serious		Not serious		0.02 (−0.10 to 0.13)		Very small	
**Resistance training**															
	4 trials (n=376)	Serious (mainly by deviations from intended interventions and missing data)		No serious (*I*^2^=16%)		Not serious		Not serious		Not serious		−0.01 (−0.07 to 0.04)		Moderate	
**Aerobic exercise**															
	3 trials (n=281)	Very serious (mainly by deviations from intended interventions and missing data)		Not serious (*I*^2^=0%)		Not serious		Not serious		Not serious		0.01 (−0.04 to 0.06)		Small	
**High-intensity interval training**															
	3 trials (n=115)	Very serious (mainly by deviations from intended interventions and missing data)		Not serious (*I*^2^=0%)		Not serious		Not serious		Not serious		0.15 (0.03 to 0.26)		Small	

^a^No: most information is from results at low risk of bias; serious: crucial limitation for one criterion or some limitations for multiple criteria sufficient to lower confidence in the estimate of effect; very serious: crucial limitation for one or more criteria sufficient to substantially lower confidence in the estimate of effect.

^b^Serious: *I*^2^>40%; very serious: *I*^2^>80%.

^c^No indirectness of evidence was found in any study.

^d^On the basis of sample size. “Serious,” n<250 participants; “very serious,” n<250 and the estimated effect is little or absent.

^e^On the basis of funnel plots. No publication bias was found. Funnel plots are not shown because the number of trials was less than 10.

^f^MD: mean difference.

## Discussion

### Principal Findings

This meta-analysis aimed to examine the effects of different types of exercise on telomere length in healthy individuals. To date, this is the only study to investigate the impact of different physical exercise modalities in a healthy population. Overall, 9 studies with 1320 participants were eligible; of them, 1299 (98%) were female participants and 91 (2%) were male participants. A total of 199 participants performed resistance exercises, 270 performed aerobic exercises, 73 performed HIIT, and 41 performed mixed exercises.

The pooled effect sizes across all telomere length outcomes showed that exercise did not significantly increase telomere length compared with the control conditions (Figure 3). This finding was robust, with little statistical heterogeneity between studies (*I*^2^=23%). Subgroup analysis suggested that HIIT was the only type of exercise that significantly increased telomere length in exercisers compared with the nonintervention group (MD 0.15, 95% CI 0.03-0.26; *P*=.01; *I*^2^=0%), with a medium effect size (standardized MD 0.41, 95% CI 0.02-0.8; *P*=.04; *I*^2^=0%). Meta-regression analyses showed that exercise intensity and duration, year of publication, and methodological quality did not influence the observed effect size. Furthermore, when we compared exercise prescription, considering intensity and duration, with LTL gain, no relationship could be established between LT and exercise intensity and duration. The 2 studies that showed the greatest improvement in LT [17,24] were not those in which the exercise prescription was more intense and longer in duration. Similarly, the methodological quality of the studies was not related to the LT gain. The study with the highest methodological quality [19] does not show a significant correlation with LT gain. Therefore, we could not establish a causal relationship between exercise prescription, methodological quality, and LT gain.

To our knowledge, this is the first study to conduct a systematic review and meta-analysis of RCTs to investigate the effects of different physical exercise modalities on telomere length in a healthy population. A recent review by Song et al [15] concluded that aerobic exercise for ≥6 months had a significant effect on the rate of telomere length shortening. However, that review included studies with heterogeneous study populations (breast cancer, polycystic ovarian syndrome, or healthy individuals). This difference in results with our study is probably because in our review, we have only included healthy populations to try to better clarify the possible impact of different physical exercises on telomere length. Telomeric shortening can be accelerated by factors that induce oxidative stress and inflammation [39]; neoplastic processes [40]; psychological disorders [41]; and chronic diseases, such as diabetes or cardiovascular disease [7,42]. Therefore, it seems clear that it is necessary to study the impact of physical exercise in specific populations because of the large number of factors that can influence telomere length.

As previously discussed, our results indicate that HIIT is the type of exercise that appears to have the most beneficial effect on LT. HIIT is characterized by short intermittent bursts of vigorous exercise interspersed with periods of low-intensity recovery [43]. This type of training has sufficient evidence to show that it is a good option for improving cardiovascular health in both healthy individuals and individuals with cardiometabolic diseases [44,45]. However, according to our results, this type of physical exercise significantly increases the length of telomeres, as intense exercise causes an increase in the total oxidative state and external production of free radicals that can lead to oxidative stress [46]. Some studies have suggested that the effects of physical exercise on LT may be represented by an inverted U-shaped dose-response [47,48]. High- or low-intensity levels (too much or too little) may have deleterious effects on the immune system and produce free radicals, thereby accelerating the aging process [49].

The different methodologies used (type of exercise and intensity), time of intervention, lack of homogeneity in the populations studied, and large number of variables that can influence LT could be the cause of the differences in the results of the different studies. Therefore, it is necessary to continue investigating the role of different modalities of physical exercise on LT in different populations by having as much control as possible over the variables that can influence telomere size in RCTs.

### Limitations and Recommendations for Future Studies

This study has several limitations. The main limitation was the small number of studies with a small sample size that performed a physical exercise intervention to assess telomere length compared to that of a control group. The included studies were heterogeneous in several aspects. The participants who underwent the interventions were healthy individuals of various ages, the vast majority of whom were women, and this might have influenced the results. The intervention protocol was heterogeneous and included exercise protocols of different intensities and application times (times/wk), some of which were incomplete. Heterogeneity was also present in the main outcome measures, showing disparities among the included studies both at baseline and postintervention measurements, although the methods used for assessing telomere length were the same.

Future research is recommended to evaluate the effects of high-intensity exercise interventions in various healthy age groups to evaluate the effect of these interventions in people with different pathologies and to establish the clinical relationship between the increase in telomere length and variables of clinical relevance.

### Recommendations for Clinical Practice

The recommendation to incorporate regular exercise, particularly through HIIT, at least 3 times a week for a sustained period, emphasizes the commitment to the preservation of health and prevention of premature aging.

### Conclusions

The findings of this systematic review and meta-analysis suggest that HIIT seems to have a positive effect on telomere length compared with other types of exercise, such as resistance training or aerobic exercise, in a healthy population. The results should be interpreted with caution because of the low quality of evidence.

## Appendix

Multimedia Appendix 1 PRISMA (Preferred Reporting Items for Systematic Review and Meta-Analyses) checklist.

Multimedia Appendix 2 Search strategy.

## Abbreviations

GRADE: Grading of Recommendations Assessment, Development, and Evaluation
HIIT: high-intensity interval training
MD: mean difference
NRSI: nonrandomized studies of interventions
RCT: randomized controlled trial
RoB2: risk-of-bias tool for randomized trials
ROBINS-I: Risk Of Bias In Nonrandomized Studies of Interventions

## Data Availability

The original contributions presented in the study are included in the article and supplementary material, and further inquiries can be directed to the corresponding author.
