# Supplementary material for: Effects of Physical Exercise on Telomere Length in Healthy Adults: Systematic Review, Meta-Analysis, and Meta-Regression
Source: JMIR Public Health Surveill. 2024 Jan 9;10:e46019. doi: 10.2196/46019 (PMC10806448; doi:10.2196/46019)
Supplement: Multimedia Appendix 2 [file publichealth_v10i1e46019_app2.docx]

| **Appendix 2:** Database formulas during literature search |
| --- |
| **PubMed Search Formula: 427**  ("telomere" OR "telomere length") AND ("physical activity" OR "exercise" OR "aerobic exercise" OR "acute exercise" OR "sports" OR "physical fitness" OR "exercise therapy" OR "resistance training" OR "muscle training" OR "exercise training" OR "physical exercise") |
| **Cochrane Library Search Formula: 111**  ("telomere" OR "telomere length") in Title Abstract Keyword AND ("physical activity" OR "exercise" OR "aerobic exercise" OR "acute exercise" OR "sports" OR "physical fitness" OR "exercise therapy" OR "resistance training" OR "muscle training" OR "exercise training" OR "physical exercise") in Title Abstract Keyword |
| **SCOPUS Search Formula: 741**  TITLE-ABS-KEY ("telomere" OR "telomere length") AND ("physical activity" OR "exercise" OR "aerobic exercise" OR "acute exercise" OR "sports" OR "physical fitness" OR "exercise therapy" OR "resistance training" OR "muscle training" OR "exercise training" OR "physical exercise") |
| **WOS Search Formula: 788** |

("telomere" OR "telomere length") AND ("physical activity" OR "exercise" OR "aerobic exercise" OR "acute exercise" OR "sports" OR "physical fitness" OR "exercise therapy" OR "resistance training" OR "muscle training" OR "exercise training" OR "physical exercise")

**Embase Search Formula: 1035**

("telomere" OR "telomere length") AND ("physical activity" OR "exercise" OR "aerobic exercise" OR "acute exercise" OR "sports" OR "physical fitness" OR "exercise therapy" OR "resistance training" OR "muscle training" OR "exercise training" OR "physical exercise")
